# Acute Stress Affects the Expression of Hippocampal Mu Oscillations in an Age-Dependent Manner

**DOI:** 10.3389/fnagi.2017.00295

**Published:** 2017-09-21

**Authors:** Samir Takillah, Jérémie Naudé, Steve Didienne, Claude Sebban, Brigitte Decros, Esther Schenker, Michael Spedding, Alexandre Mourot, Jean Mariani, Philippe Faure

**Affiliations:** ^1^Team Neurophysiology and Behavior, Institut de Biologie Paris Seine (IBPS), UMR 8246 Neuroscience Paris Seine (NPS), Sorbonne Universités, Université Pierre et Marie Curie (UPMC), CNRS, INSERM, U1130 Paris, France; ^2^Team Brain Development, Repair and Ageing, Institut de Biologie Paris Seine (IBPS), UMR 8256 Biological Adaptation and Ageing (B2A), Sorbonne Universités, Université Pierre et Marie Curie (UPMC), CNRS Paris, France; ^3^APHP Hôpital Charles Foix, DHU Fast, Institut de la Longévité Ivry-sur-Seine, France; ^4^Département Neurosciences et Contraintes Opérationnelles, Institut de Recherche Biomédicale des Armées (IRBA), Unité Fatigue et Vigilance Brétigny-sur-Orge, France; ^5^EA7330 VIFASOM, Université Paris Descartes Paris, France; ^6^Neuroscience Drug Discovery Unit, Institut de Recherches Servier Croissy-sur-Seine, France; ^7^Spedding Research Solutions SARL Le Vésinet, France

**Keywords:** aging, stress, hippocampus, Mu-rhythm, synchronized oscillation

## Abstract

Anxiolytic drugs are widely used in the elderly, a population particularly sensitive to stress. Stress, aging and anxiolytics all affect low-frequency oscillations in the hippocampus and prefrontal cortex (PFC) independently, but the interactions between these factors remain unclear. Here, we compared the effects of stress (elevated platform, EP) and anxiolytics (diazepam, DZP) on extracellular field potentials (EFP) in the PFC, parietal cortex and hippocampus (dorsal and ventral parts) of adult (8 months) and aged (18 months) Wistar rats. A potential source of confusion in the experimental studies in rodents comes from locomotion-related theta (6–12 Hz) oscillations, which may overshadow the direct effects of anxiety on low-frequency and especially on the high-amplitude oscillations in the Mu range (7–12 Hz), related to arousal. Animals were restrained to avoid any confound and isolate the direct effects of stress from theta oscillations related to stress-induced locomotion. We identified transient, high-amplitude oscillations in the 7–12 Hz range (“Mu-bursts”) in the PFC, parietal cortex and only in the dorsal part of hippocampus. At rest, aged rats displayed more Mu-bursts than adults. Stress acted differently on Mu-bursts depending on age: it increases vs. decreases burst, in adult and aged animals, respectively. In contrast DZP (1 mg/kg) acted the same way in stressed adult and age animal: it decreased the occurrence of Mu-bursts, as well as their co-occurrence. This is consistent with DZP acting as a positive allosteric modulator of GABA_A_ receptors, which globally potentiates inhibition and has anxiolytic effects. Overall, the effect of benzodiazepines on stressed animals was to restore Mu burst activity in adults but to strongly diminish them in aged rats. This work suggests Mu-bursts as a neural marker to study the impact of stress and DZP on age.

## Introduction

Stress is a set of physiological responses triggered by an aversive situation (Kim and Diamond, 2002). It is generally associated with anxiety disorder (Pêgo et al., 2008; Bessa et al., 2009), a state characterized by “hypervigilance” (i.e., a high level of arousal) and sustained alertness for potential threats (Sylvers et al., 2011; Adhikari, 2014; Tovote et al., 2015). Stress also promotes avoidance and is often associated with fear generalization (Duvarci et al., 2009; Davis et al., 2010). A key point is that reaction to stress is strongly age-dependent, with elderly people enduring stressful situations more frequently and reacting to pressure more profoundly (Prenderville et al., 2015). In particular, aging may induce sustained stress reactions (Wikinski et al., 2001; Leite-Almeida et al., 2009; Pietrelli et al., 2012). The neurological consequences of stress and age appear furthermore strikingly similar: both are associated with alterations of neuronal plasticity and increased risk of brain disorders (Morrison and Baxter, 2012; Prenderville et al., 2015). These similarities suggest that age itself may act as a stressor factor (Buechel et al., 2014). This link between age and stress is highlighted by an altered brain plasticity in elderly after exposure to new-onset stress (Morrison and Baxter, 2012; Lindenberger, 2014; Prenderville et al., 2015), and that aged individuals often cope with stressful situations (Barrientos et al., 2012; Buechel et al., 2014).

A proper understanding of the interactions between age and stress is crucial when considering the wide use of anxiolytic drugs such as benzodiazepines in the elderly (Gleason et al., 1998; Kirby et al., 1999). Benzodiazepines like diazepam (DZP) have a number of clinically approved uses (reduction of sleep latency, muscle relaxation, anxiolysis…) but also have unwanted side effects, in particular a decreased alertness, anterograde amnesia, dependence and addiction (Tan et al., 2011). Benzodiazepines influence behavioral activity and, accordingly, neural oscillations in cortical circuits. DZP is a positive allosteric modulator of the GABA_A_ receptor that acts by potentiating the natural ligand GABA (Tan et al., 2011). At the synaptic level, DZP enhances the amplitude and duration of inhibitory postsynaptic events, and thus increases phasic inhibition (Scheffzük et al., 2013). At the network level, this potentiation of inhibition results in characteristic alterations of rhythmic activity patterns (Dimpfel et al., 1988; van Lier et al., 2004; Botta et al., 2015).

The organized activity of neural networks, as reflected in multi-neuronal, extracellular fields potential (EFP) recordings, frequently presents a rhythmic quality. In humans, the central 7–12 Hz rhythm, also called Mu-rhythm in the sensorimotor/parietal area, reflects an idling state (Gastaut et al., 1965). This oscillatory index, characterized by bursts of oscillations of high amplitude, has been mostly observed in somatosensory cortex and is known to be modulated by attention (Wiest and Nicolelis, 2003; Fontanini and Katz, 2005; Tort et al., 2010; Coll et al., 2017). However, other studies found Mu oscillations in many fronto-parietal regions (Sakata et al., 2005; Marini et al., 2008; Tort et al., 2010) and even in the cerebellum (Hartmann and Bower, 1998). In rats, the role of 7–12 Hz cortical rhythm remains a topic of intense debate (Nicolelis et al., 1995; Nicolelis and Fanselow, 2002; Wiest and Nicolelis, 2003; Shaw, 2004, 2007; Fontanini and Katz, 2005). It has been proposed to represent a dynamical filter for detecting weak or novel tactile stimuli (Wiest and Nicolelis, 2003) or a withdrawal state (i.e., with internally-directed attention; Fontanini and Katz, 2005). This rhythm is associated with whisker twitching (WT), during which rats stand still and twitch their whiskers in small-amplitude movements, inducing an increase of sensitivity to weak sensory signals (Nicolelis et al., 1995; Fanselow et al., 2001). However, it remains unknown whether stressful situations can switch the vigilance state towards such quiet alertness, reflected by an increased occurrence of the 7–12 Hz oscillations.

In this study, we recorded EFP in the dorsal and ventral hippocampus (d/v-HPC), prefrontal cortex (PFC) and parietal associative cortex (PAR, formerly called sensorimotor cortex in the somatosensory system) in adult and aged rats, at rest (control) and on an elevated platform (EP; stress condition), with systemic injections of saline or DZP, in order to assess the interactions between stress, age and anxiolytics on alertness-related cortical rhythms. We show that stress increased the 7–12 Hz rhythms of the PFC, PAR and HPC in adult rats but that, inversely, it decreased these rhythms in aged rats. Furthermore, we reveal an interaction between DZP, age and stress that may bear important implications for the anxiolytic effects of DZP in the elderly.

## Materials and Methods

### Animals Care, Housing Conditions and Ethics Statement

All experiments were approved by the Ethic committee CAPSUD/N°26 (Ministère de l’Enseignement Supérieur et de la Recherche, France) and conducted in agreement with institutional guidelines and in compliance with national and European laws and policies (Project no. 01272.01). Experiments were performed on 17 adults (8 months) and 10 aged (strictly speaking, late middle-aged rats, 18 months (Prenderville et al., 2015), but referred to here as “aged”) male Wistar rats from Janvier Laboratories. The animals were singly housed, in a 12 h light/dark cycle and temperature-controlled room (22 ± 2°C) with food and water available *ad libitum*.

### *In Vivo* Electrophysiological Recordings

Rats were anesthetized with ketamine and xylazine and placed in a stereotaxic frame. Anesthesia was maintained with inhalation of a mixture of isoflurane 3% and oxygen. Bipolar stainless steel electrodes were chronically implanted bilaterally in each rat into the infralimbic/prelimbic of PFC, the PAR, the CA1 dHPC and the CA1 vHPC. Monopolar ground electrodes were laid over the cortical layer of the cerebellum and the olfactory bulb. Electrodes were connected to an electrode interface board (QuickClip Connect EIB-16-QC-H, Neuralynx) and dental acrylic was used to fix them to the skull during the surgery. Six bipolar electrodes (the distance between the recording tips and the reference tips were 0.7 mm for the PFC or PAR and 0.5 mm for the different part of the HPC) were implanted through burr holes targeting the following coordinates from Bregma: depth over the cortex 3.8 mm, AP +3 mm, ML ± 0.8 mm for the PFC; depth over the cortex 0.7 mm, AP −4 mm, ML ± 4 mm for PAR; depth 2.8 mm, AP −3.6 mm, ML ± 2.2 mm for the dHPC; depth over the cortex 5.3 mm, AP −6.3 mm, ML ± 5.6 mm for the vHPC. The recording tips were located in the deep layers and the local reference tips at the surface of the corresponding cortices.

Finally, to reduce electrical noise, two grounds (monopolar electrodes) were implanted over the cortex at the following coordinates from Bregma, AP +6.7 mm, ML ± 1 mm for the olfactory bulb; AP −11 mm, ML ± 1 mm for the cerebellum (Paxinos and Watson, 2006).

After surgery, an antiseptic (Povidone-iodine solution) and a local anesthetic (lidocaine ointment) were applied in all areas where the scalp had been incised. Animals were permitted to recover until regaining pre-surgery body weight.

### Protocol Design

Experimental setting is based on previous studies (Sebban et al., 1999a,b, 2002). EFP obtained from the dHPC generally exhibit prominent theta-frequency oscillations. Two types of hippocampal theta activity were described in the rat. One type was termed atropine-sensitive theta, since it was abolished by the administration of atropine. Atropine-sensitive theta occurred during immobility in rodents in the normal state. The other type of theta was termed atropine-resistant, since it was not sensitive to treatment with atropine but was abolished by locomotors activities or anesthetics. Atropine-sensitive theta became known as type II (immobility-related) theta. Atropine-resistant theta became known as type I theta, since it occurred during Type I (voluntary) motor behaviors, such as walking, rearing and postural adjustments. These oscillations can be modulated by stress, in the case of type II theta, related to cognition (Hsiao et al., 2012) but also by other behavioral variables, in particular by locomotion, in the case of type I theta (Vanderwolf, 1969; Buzsáki, 2002). In the theta frequency range, Mu oscillations are thought to reflect the vigilance state (Kramis et al., 1975; Sakata et al., 2005; Popa et al., 2010) but can be affected by sensory inputs (Fanselow et al., 2001; Tort et al., 2010; Aitake et al., 2011; Fries, 2015). We thus aimed at eliminating potential confounds related to type I theta and sensory influences on Mu oscillations. To minimize the EFP modulations induced by spontaneous locomotor activity (type I theta), rats were restrained in a resting-state environment box and were gradually accustomed to be restricted in their movements, 10 min the first day and with an additional 10 min every day (D1: 10 min, D2: 20 min, D3: 30 min…), until the recording time was reached. This procedure required approximatively 10–14 days for each animal to remain quiet. Furthermore, a cold light source of 100 lux was applied at a distance of 10 cm in front of the rat’s nose to keep the animal still, with the head up (the animal voluntarily kept the head up due to the light source) and wide-open eyes (Sebban et al., 1999a,b). During recordings, rats were isolated into a large, electrically and acoustically insulated chamber, in a specific recording room, to eliminate as far as possible to any sensory input that might affect the Mu oscillations.

Recordings were obtained using a Digital Lynx SX (Neuralynx). Sixty minutes baseline EFP recordings were obtained while the animals remained relatively still. The effect of stress was evaluated 1 day later by placing rats on an EP (Xu et al., 1998; Rocher et al., 2004) of small size in the same experimental room and condition. To avoid any bias linked to circadian variation of EFP (Sebban et al., 2002) both recording sessions took place exactly at the same hour of the day. Adult and aged rats were examined simultaneously excepted for five additional adult rats. We checked that the restraining procedure do not produce stress symptoms by itself: no attempts to escape or notable stress reactions were observed (i.e., defecation, urination, freezing) at rest at the end of the habituation procedure, contrarily to the stress condition. Throughout all the recording, rats showed quick reactions when probed by slight sound stimulation, by turning their head toward the sound. We did not observe any difference in the propensity to detect the sound, nor in the reaction times between adult and aged rats, but no quantitative comparison of sensory functions between adult and aged rats were performed.

### Neurophysiological Data Analysis

Data were analyzed using Matlab (Matworks^®^) built-in and custom-written codes. All EFP: (i) were acquired at 1000 Hz and offline band-pass filtered between 0.1 Hz and 100 Hz with a zero-phase shift filter function (zero-phase digital filtering *filtfilt* function); (ii) detrended using local linear regression (*locdetrend* function from the Chronux toolbox; Bokil et al., 2010): window size 1 s, overlap 0.5 s) to remove slow drifts; and (iii) notch-filtered (*iirnotch* function), with the notch located at 50 Hz to remove any possible power line noise. EFP signal was expressed in z-score units. The z-score normalization used the mean and the standard deviation from the baseline (entire rest session) of each electrode. Multitaper *spectrogram* method from the Chronux toolbox (Bokil et al., 2010) with time-bandwidth product of 5 and 10 slepian sequences of orthogonal data tapers was used to calculate power spectral density (PSD) of the EFP data, using a window size of 5 s, with 2 s overlap. PSD was averaged over two similar brain regions (right and left hemisphere) in each animal, for each frequency and time bin. The multitaper *coherogram* method was used to calculate the coherence (normalized spectral covariance) between the EFP from two structures with time-bandwidth product of 30 and 60 slepian sequences of orthogonal data tapers, using a windows size of 30 s without overlap. The signal was bandpass-filtered to extract Mu-oscillations by applying a 7–12 Hz finite impulse response (FIR) bandpass with zero-phase shift filter function (*filtfilt* function).

Instantaneous amplitude and phase from the EFP were obtained using a continuous Morlet wavelet transform, with matcher filter construct parameters: center frequency = 1 and bandwidth = 2, for the 0.1–30 Hz range. Wavelet coherence was computed by smoothing the product of the two relevant wavelet transforms over time (window for time smoothing = 0.6 s) and over scale (pseudo-frequency) steps (window for scale smoothing = 3 Hz).

We measured the phase locking value (as an index for synchrony) between EFP in the PFC and dHPC from the wavelet coherence, using the distribution of the phase differences between EFPs (Lachaux et al., 2002). This measurement is a normalized index of the stability of phase shifts which varies between 0 (random distribution, no phase synchrony) and 1 (perfect phase synchrony locking; Le Van Quyen et al., 2001).

The international classification of the borders between the different frequency bands was arbitrarily drawn (Delta, 0.5–4 Hz; Theta 4–8 Hz; Alpha, 8–12 Hz; Beta, 12–30 Hz; Gamma >30 Hz). In the freely moving rodent, hippocampal theta should be designated theta-alpha, according to the committee’s recommendation, since theta varies between 6–7 Hz and 12 Hz (Vanderwolf, 1969; Winson, 1978; Bland, 1986; Buzsáki, 2002; Yamamoto et al., 2014). Hence, we did not separate alpha from theta and considered the whole 7–12 Hz range for statistical testing. A great variety of rhythms in the same 7–12 Hz range have been described in the thalamocortical system, including Mu rhythm (8–12 Hz, sensorimotor system), together with alpha waves (8–12 Hz, visual system), tau rhythms (8–12 Hz, auditory system) or sleep spindles (10–20 Hz). Hence, in rodents, Mu and Theta oscillations share the same frequency component. However, they differ in their voltage intensity i.e., Mu exhibiting higher amplitude of oscillations. These particularly large amplitude oscillations makes sometimes called high-voltage spindle or spike-and-wave discharge (Robinson and Gilmore, 1980; Inoue et al., 1990; Shaw, 2007). We thus separated Mu oscillations from type II Theta rhythm by an amplitude threshold, with the highest voltage events classified as Mu and residual activity considered as theta, with the following procedure. We measured the amplitude in the 7–12 Hz range from the area under the curve (AUC; *trapz* function) of the complex Morlet wavelet transform in this range. Finally, Mu-bursts were extracted by: (i) smoothing the filtered power of 7–12 Hz wavelet transform with a Kalman filter; and (ii) using a double threshold (for the beginning and end of a burst) and a persistence greater than 3 s, i.e., Mu-burst started when the EFP was above the upper thresholds for more than 3 s, and ended after switching below the lowest threshold for a duration greater than 3 s.

### Drugs Preparation and Pharmacological Protocol

DZP (Sigma-Aldrich), a standard anxiolytic in humans and rodents (van Lier et al., 2004; Scheffzük et al., 2013), was prepared in a 10% 2-hydroxypropyl-β-cyclodextrin solution and saline. The same solvent was used as vehicle in control experiments. DZP was injected intra-peritoneally at a single dose of 1 mg/kg, which is known to exert an anxiolytic effect. Higher doses were not tested as they may induce sedation (Wikinski et al., 2001; van Lier et al., 2004). Recordings started immediately after DZP administration. Five days before experiments, rats were daily prepared for intra-peritoneal administration by exerting a light pressure on the body with a syringe. The effect of DZP vs. vehicle administration was evaluated in the two conditions, i.e., at rest and under stress (in the EP), for 145 min. Moreover, to avoid the bias linked to circadian variation of EFP, both recording sessions took place exactly at the same hour. The rats were randomly assigned to a given treatment according to a within-subject “Latin square”. A free week was imposed between two interventions.

### Statistical Analysis

All datasets were tested for normality using Shapiro-Wilk and Lilliefors tests. For statistical comparison, three bands of the PSD were analyzed for each structure: 0.1–4 Hz (Delta), 7–12 Hz (Mu) and 12–30 Hz (Beta). No statistical methods were used to predetermine sample sizes, but our sample sizes are similar to those reported in previous publications.

For single comparisons, paired-sample *T*-tests (normally distributed data) or non-parametric Wilcoxon’s signed rank tests (non-Gaussian distribution or for small samples) were used to compare PSD and coherence estimates of the Mu-rhythm from the same animals. Two-sample *T*-tests or nonparametric Wilcoxon’s rank-sum tests were used to compare PSD and coherence estimates between adult and aged groups.

For multiple comparisons of normally distributed data, we used one-way ANOVA (e.g., adults/aged rats) or two-way ANOVA (e.g., with frequency bands and stress/rest as factors). For data with non-Gaussian distribution or for small samples, non-parametric tests were used: Kruskal-Wallis test (instead of one-way ANOVA) and Friedman test (instead of two-way ANOVA). *Post hoc* tests were performed to identify which frequency bands differed in the spectral analysis and which groups differed in the wavelet-transform analysis. We did not compare different frequency bands from different conditions (e.g., theta in adults with delta in aged rats). We used respectively the stepwise algorithms Holm-Bonferroni to correct family-wise error rate (i.e., potential interferences during multiple comparisons) by ordering the *p*-values and adjusting the significant level α and Tukey’s honest significant difference (HSD) criterion.

Results are expressed as mean ± standard error of the mean (SEM) and were listed in table. SEM intervals were calculated through a jackknife method (Bokil et al., 2010). The level of statistical significance was set at 5% for all tests (two-sided). Significance levels are shown in figures with one to three asterisks (*p < 0.05, **p < 0.005, ***p < 0.0005).

## Results

### Electrophysiological Signatures of Stress

**Figure 1:**
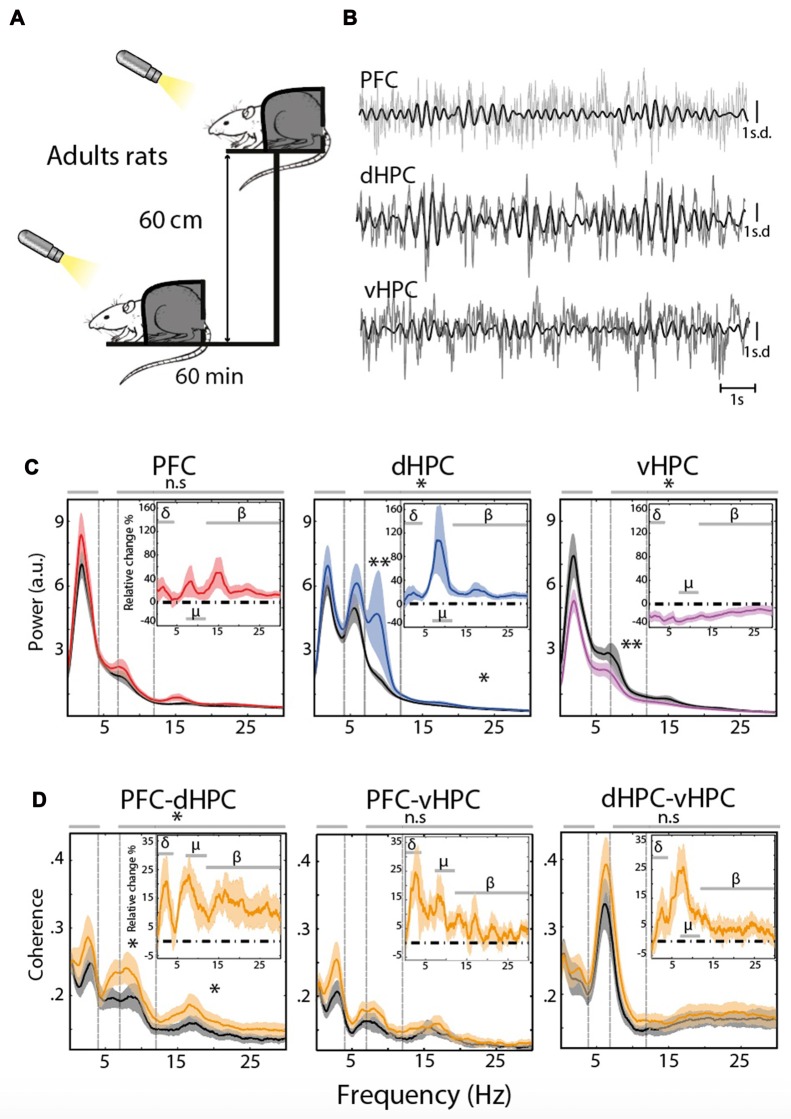
**(A)** Stress protocol: 60 min at rest, followed 1 day later by 60 min under stress on an elevated platform (EP). A cold light source (100 lux) was applied at a distance of 10 cm in front of the rat’s nose to keep the eyes of the animal wide open and its head held up. **(B)** Representative traces of the Z-scored extracellular fields potential (EFP) simultaneously recorded from the same animal in the prefrontal cortex (PFC), dorsal hippocampus (dHPC) and ventral hippocampus (vHPC) at rest. Raw traces are plotted in gray and filtered (7–12 Hz range) traces are overlaid in black. **(C)** Spectral analysis of the EFP recorded at rest for each structure (black) and under acute stress (red: PFC *n* = 13, blue: dHPC *n* = 13, purple: vHPC *n* = 12). The top right insert represents the averaged relative change, expressed in percentage of variation. Horizontal dashed line at zero indicates no change. Data are presented as mean ± standard error of the mean (SEM) and shaded area indicates SEM. **(D)** Coherence for PFC-dHPC, PFC-vHPC and dHPC-vHPC at rest (black) and under stress (orange). In top right insert, averaged relative change expressed in percentage of variation. Horizontal dashed line at zero indicates no change. Data are presented as mean ± SEM and shaded area indicates SEM.

Electrophysiological signatures of stress-induced activities in the PFC and HPC were evaluated by comparing PSD of EFP obtained for adult rats in a state of quiet wakefulness at rest, or in a stressful condition when animals were placed on an EP (Figures 1A,B). Restraining rats allowed to avoid theta oscillations related to locomotion (type I theta, prominent in the dHPC, see “Materials and Methods” Section) and thus to correctly evaluate the impact of acute stress on EFP frequency content, and in particular type II theta and Mu oscillations, which are both related to arousal and vigilance (Kramis et al., 1975; Shaw, 2004; Tort et al., 2010; Sobolewski et al., 2011; Wells et al., 2013).

Comparison of EFP content in specific frequency bands (i.e., delta (0.1–4 Hz), Mu (7–12 Hz) and beta (12–30 Hz), see Table 1) showed a significant increase in the Mu and Beta ranges of the dHPC EFP (*n* = 13, χ^2^ = 4.36; *P = 0.0369 Friedman’s test followed by Wilcoxon’s signed rank-test **P_mu_ = 7.3242e-4, and paired sample *T*-test *P_beta_ = 0.0188, Figure 1C, middle). In contrast, the PSD of vHPC (*n* = 12) was globally reduced following the stress procedure, in particular in the Mu range (*F*_(1,66)_ = 7.04; *P = 0.01 Two-way ANOVA test followed by Wilcoxon’s signed-rank test **P_mu_ = 4.8828e-4, Figure 1C, right).

**Table 1:** Interactions of age and stress on prefrontal cortex (PFC) and hippocampus (HPC) oscillations.

Group	Figure	Test	*P* value	*Post hoc*	Correction	*P* value
**Adults**: Rest/Stress **PFC**: *n* = 13	1C Left	Two-way ANOVA (condition × bands)	*F*_(1,72)_ = 119.11; *P* = 0.1719 (condition)	Not applicable (N/A)	N/A	N/A
**Adults**: Rest/Stress **dHPC**: *n* = 13	1C Middle	Friedman ANOVA Table (condition × bands)	χ^2^ = 4.36; *P = 0.0369 (condition)	Delta: Wilcoxon signed rank test Mu: Wilcoxon signed rank test Beta: Paired sample *t*-test	Holm-Bonferroni: α< 0.05 α/3< 0.0167 α/2< 0.025	n/s *P*_Delta_: 0.1188 ***P_Mu_: 7.3242e-4 *P_Beta_: 0.0188
**Adults**: Rest/Stress **vHPC:** *n* = 12	1C Right	Two-way ANOVA (condition × bands)	*F*_(1,66)_ = 7.04; *P = 0.01 (condition)	Delta: Paired sample *t*-test Mu: Wilcoxon signed rank test Beta: Paired sample *t*-test	Holm-Bonferroni: α/2< 0.025 α/3< 0.0167 α< 0.05	n/s *P*_Delta_: 0.0336 ***P_Mu_: 4.8828e-4 n/s *P*_Beta_: 0.0345
**Adults**: Rest/Stress **Coherence_PFC-dHPC_** *n* = 13	1D Left	Friedman ANOVA Table (condition × bands)	χ^2^ = 4.61; *P = 0.0318 (condition)	Delta: Wilcoxon signed rank test Mu: Paired sample *t*-test Beta: Wilcoxon signed rank test	Holm-Bonferroni: α< 0.05 α/3< 0.0167 α/2< 0.025	n/s *P*_Delta_: 0.2439 *P_Mu_: 0.0076 *P_Beta_: 0.0085
**Adults**: Rest/Stress **Coherence_PFC-vHPC_** *n* = 12	1D Middle	Two-way ANOVA (condition × bands)	*F*_(1,60)_ = 3.98; *P* = 0.0507 (condition)	N/A	N/A	N/A
**Adults**: Rest/Stress **Coherence_dHPC-vHPC_** *n* = 12	1D Right	Two-way ANOVA (condition × bands)	*F*_(1,60)_ = 1.31; *P* = 0.2566 (condition)	N/A	N/A	N/A
**Aged**: Rest/Stress **PFC**: *n* = 10	5C Left insert	Friedman ANOVA Table (condition × bands)	χ^2^ = 5.15, *P = 0.0232 (condition)	Delta: Paired sample *T*-test Mu: Wilcoxon signed rank test Beta: Wilcoxon signed rank test	Holm-Bonferroni: α/3< 0.0167 α/2< 0.025 α< 0.05	n/s *P*_Delta_: 0.0495 n/s *P*_Mu_: 0.3750 n/s *P*_Beta_: 0.4316
**Aged**: Rest/Stress **dHPC**: *n* = 10	5C Middle insert	Friedman ANOVA Table (ages × bands)	χ^2^ = 15.61, ***P = 7.781e-5 (condition)	Delta: Wilcoxon signed rank test Mu: Paired sample *t*-test Beta: Paired sample *t*-test	Holm-Bonferroni: α/3< 0.0167 α< 0.05 α/2< 0.025	*P_Delta_: 0.0078 *P_Mu_: 0.0291 *P_Beta_: 0.0118
**Aged**: Rest/Stress **vHPC:** *n* = 5	5C Right insert	Friedman ANOVA Table (condition × bands)	χ^2^ = 5.53, *P = 0.0187 (condition)	Delta: Paired sample *t*-test Mu: Wilcoxon signed rank test Beta: Paired sample *t*-test	Holm-Bonferroni: α/2< 0.025 α< 0.05 α/3< 0.0167	n/s *P*_Delta_: 0.1866 n/s *P*_Mu_: 0.1875 n/s *P*_Beta_: 0.1343
**Aged**: Rest/Stress **Coherence_PFC-dHPC_** *n* = 10	5D Left insert	Friedman ANOVA Table (condition × bands)	χ^2^ = 5.76, *P = 0.0164 (condition)	Delta: Paired sample *t*-test Mu: Wilcoxon signed rank test Beta: Wilcoxon signed rank test	Holm-Bonferroni: α< 0.05 α/3< 0.0167 α/2< 0.025	*P_Delta_: 0.0166 *P_Mu_: 0.0098 *P_Beta_: 0.0137
**Aged**: Rest/Stress **Coherence_PFC-vHPC_** *n* = 5	5D Middle insert	Friedman ANOVA Table (condition × bands)	χ^2^ = 4.98, *P = 0.0257 (condition)	Delta: Paired sample *t*-test Mu: Wilcoxon signed rank test Beta: Wilcoxon signed rank test	Holm-Bonferroni: α/3< 0.0167 α< 0.05 α/2< 0.025	*P_Delta_: 0.0850 *P_Mu_: 0.4375 *P_Beta_: 0.2911
**Aged**: Rest/Stress **Coherence_dHPC-vHPC_** *n* = 5	5D Right insert	Two-way ANOVA ANOVA Table (condition × bands)	*F*_(1,24)_ = 0.02, *P* = 0.8766 (condition)	N/A	N/A	N/A

For multicomparisons tests (Two-way ANOVA or Friedman), significant levels (statistical significance was set at 5%) are presented with one to three asterisks (*P < 0.05, **P < 0.005, ***P < 0.0005) and for post hoc tests (Wilcoxon signed rank test or paired sample *t*-test) i.e. multicomparisons followed by Holm-Bonferroni corrections, by ordering the *p*-values *p*(1) < *p*(2) < *p*(3) and adjusting the significant level (1) α/3 < 0.0167,(2) α/2 < 0.025, (3) α < 0.05.

These differences in EFP co-occurred with a modification of the functional connectivity between brain areas. This functional connectivity was evaluated using pairwise coherences (i.e., co-modulation in amplitude and phase-shift stability between two structures) between the PFC, dHPC and vHPC (Figure 1D). At rest, coherence in the delta/Mu ranges was relatively higher between the ventral and dorsal HPC than between the PFC and either part of the hippocampus (Figure 1D). The stress protocol induced only a clear increase in coherence between the PFC and dHPC in the Mu and beta ranges (Figure 1D left (χ^2^ = 4.61; *P = 0.0318 Friedman’s test followed by paired *T*-test: *P_mu_ = 0.0076 and Wilcoxon’s signed-rank test: *P_beta_ = 0.0085). Eliminating potential locomotion-related effects thus revealed that stress increased both the amplitude of the Mu rhythm in the dHPC and its coherence with mPFC in adult rats.

### Stress-Induced Modifications of Mu Rhythms Are Composed of Mu-Bursts of Oscillations, Associated with Whisker Twitching and Alertness

**Figure 2:**
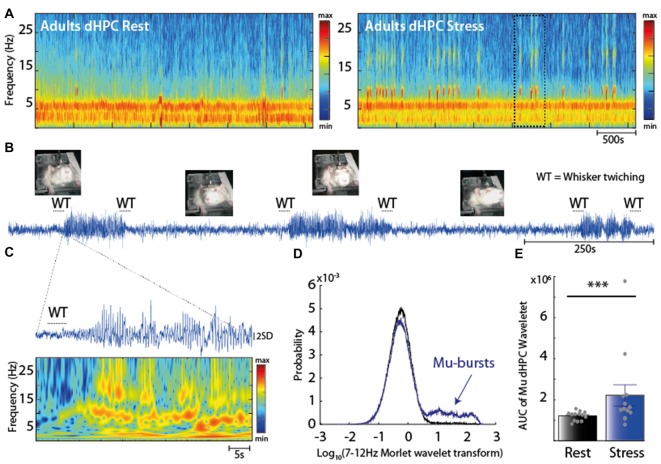
**(A)** Spectrogram of dHPC at rest (left panel) and under stress (right panel). Note the emergence of intermittent oscillations in the 7–12 Hz range in stress condition. **(B)** Raw trace of the dHPC and its behavioral correlate. Note the prominent increase in the raw signal. Most of the Mu-bursts events were associated with whisker twitching (WT) both at the onset and ending. **(C)** EFP Z-scored trace and time-resolved power spectral density (PSD; using a complex Morlet wavelet transform) during a Mu-burst. Mu-bursts correspond to an oscillation with a dominant frequency peak around 7–12 Hz, together with one to several biological harmonics. **(D)** Distribution of 7–12 Hz power across time at rest (black) and under stress (blue). The bell curve of the distribution after a log transform reveals a unimodal distribution. Note this another peak appearing under stress, with the rest of the distribution unchanged, corresponding to the Mu-bursts associated with WT. **(E)** Area under the curve (AUC) computed from the Morlet wavelet transform (averaged over the 7–12 Hz range) that reflects the overall amplitude of Mu-bursts. The Mu-bursts AUC increased significantly in stress condition (***p < 0.001, *n* = 13).

We next investigated whether the stress-induced increase in the dHPC Mu rhythm was related to a state of alertness, and thus could reflect an effect of stress on vigilance. Indeed, the time-dependent spectrogram of the dHPC EFP revealed transient bursts of oscillations in the Mu range (Figure 2A), thereafter called Mu-bursts, which were observed in every rat. While they rarely occurred in adult rats at rest (Figure 2A, left), their occurrence increased under the stress condition (Figure 2A, right). Mu-bursts were almost systematically associated with an exploratory behavior of “WT” (Figure 2B), i.e., an alert state where rats are still, keep their eyes open, and twitch their whiskers in rhythmic, small-amplitude movements (Fanselow et al., 2001; Sobolewski et al., 2011). This contrasted with the usual behavioral pattern at rest, where rats moved their head left and right, without rhythmic whisker movements. A careful examination of the time-resolved power (of dHPC EFP) obtained from the Morlet wavelet transforms revealed that Mu-bursts were composed of two main frequency contents: one between 7 Hz and 12 Hz, and one at higher frequencies possibly reflecting a “biological harmonic” (Figure 2C). PSD obtained from wavelets transforms of the dHPC EFP in the 7–12 Hz range at rest followed a unimodal distribution (Figure 2D, black curve), as commonly found throughout cortices (Roberts et al., 2015). This type of distribution indicates that synchronous events, i.e., high-amplitude oscillations, were irregularly interspersed with smaller-sized events. Nevertheless, in the stressful situation, another peak appeared in this distribution for large PSD values, while the rest of the distribution was unchanged (Figure 2D, blue). Hence Mu-bursts did not reflect an overall increase in oscillation amplitude, which would have resulted in a rightward shift of the distribution. Rather, they constituted discrete events that were clearly distinct from baseline oscillations and that co-occurred with WT. Furthermore, analysis of the AUC of the wavelet PSD confirmed an increase in the 7–12 Hz oscillations under stress condition (***p = 5e-4, Wilcoxon’ sign rank test Figure 2E). Overall the increase in the number of Mu-bursts recapitulated the increase in dHPC Mu band induced by stress.

### Stress-Induced Mu-Bursts Co-Occur in the dHPC and PFC

**Figure 3:**
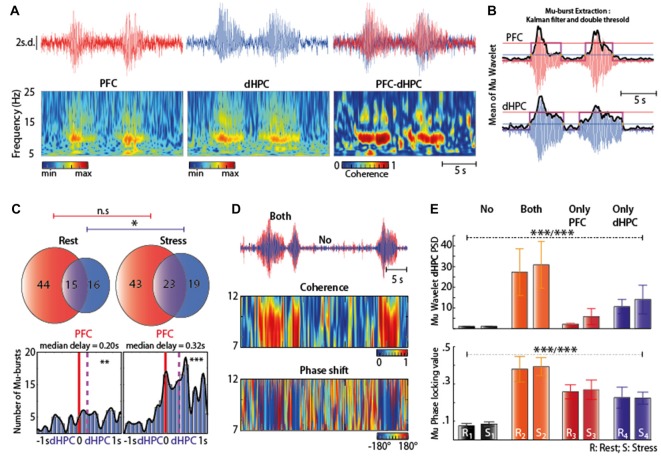
**(A)** Typical examples of Mu-bursts in Z-scored EFP traces (top) and corresponding time-resolved PSD (bottom) in both the PFC (left) and dHPC (middle), as well as the superposition of these traces (top right) and the time-resolved coherence between the PFC and dHPC (bottom right). These Mu-bursts consisted in oscillations at the same frequencies (7–12 Hz). **(B)** Extraction of discrete Mu-bursts: (i) raw EFP (red: PFC; blue: dHPC) was wavelet-transformed in the 7–12 Hz range, averaged over frequency and smoothed across time (Kalman filter), resulting in the black trace. Mu-bursts starts and stops were determined using a double threshold, one for the onset (blue line) and another for the completion (red line), also constrained by a burst duration greater than 3 s. **(C)** Top: Venn diagram illustrating the average occurrence of Mu-bursts in each structure and their co-occurrence; Bottom: distribution of time lags between Mu-bursts onsets, from dHPC relative to PFC. Red line corresponds to zero-lag and purple line represents the median lag. **(D)** Top: superimposed EFP (red: PFC; blue: dHPC) showing both epochs of Mu-bursts and of baseline oscillations. Middle: time-resolved coherence between the PFC and dHPC. Coherence is maximal during Mu-bursts. Bottom: difference in instantaneous phases from the wavelet transforms (phase-shift) of dHPC and PFC, indicating phase-locking during Mu-bursts. **(E)** Top: square of the absolute value of the wavelet transform when no Mu-bursts occur (“No”), Mu-bursts occur in both the PFC and dHPC (“both”) and only in one of the two structures (“Only PFC”, “Only dHPC”) in two condition (R: rest; S: stress), (***P_rest_/***P_stress_) Tukey’s honest significant difference (HSD) test indicated statistical difference between the following pairs: (Rest: R_1_-{R_2_, R_4_}; R_2_-{R_1_}; R_3_-{naught}; R_4_-{R_1_}; Stress: S_1_-{S_2_, S_4_}; S_2_-{S_1_, S_3_}; S_3_-{S_2_}; S_4_-{S_1_}). Bottom: phase-locking value), (***P_rest_/***P_stress_). See Table 2which indicated statistical difference between pairs.

**Figure 4:**
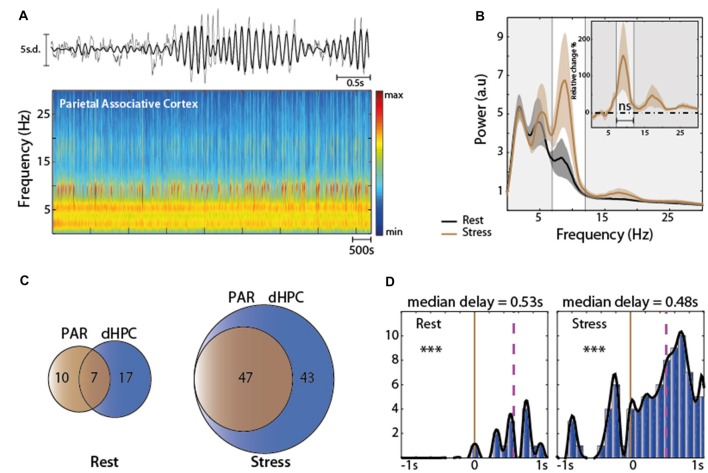
**(A)** Top: representative traces of the Z-scored EFP in the parietal associative cortex (PAR). Raw traces are plotted in gray and filtered (7–12 Hz range) traces are overlaid in black; Bottom: spectrogram of PAR under stress. **(B)** Spectral analysis of the EFP recorded at rest structure (black) and under acute stress (brown) *N* = 5. **(C)** Venn diagram illustrating the average occurrence of Mu-bursts in dHPC and PAR and their co-occurrence. Note at rest, Mu-burst are observed independently in the two neighboring structures. Under stress condition the total number increased in both structure. Note that all Mu-bursts detected in PAR co-occurred in the dHPC. **(D)** Distribution of time lags between Mu-bursts onsets, from dHPC relative to PAR. Brown line corresponds to zero-lag and purple line represents the median lag. Note that all Mu-bursts detected in PAR co-occurred in the dHPC. Note that the Mu-burst are observed independently in the two neighboring structures. Rest: 0.53 s median delay, paired sample *T*-test ***P < 0.001 stress: 0.48 s median delay, Wilcoxon signed rank test ***P < 0.001.

We next assessed whether the Mu-bursts observed in the HPC were correlated with similar activity in the PFC. Mu-burst were indeed detected both in the PFC, dHPC (Figure 3A, left-middle) and in the parietal cortex (Figure 4), but not in the vHPC. Coherence between the dHPC and PFC was maximal during the Mu-bursts (Figure 3A, right), which may explain why coherence increases during stress (see Figure 1D, left). Individual detection of Mu-bursts (see “Materials and Methods” Section, Figure 3B) indicated that they occurred more often in the PFC than in the dHPC, both at rest and under stress (Figure 3C, top). At rest, about half (56% ± 16, mean ± SEM) of the dHPC Mu-bursts appeared concomitantly in the PFC, while one fourth (26% ± 10, mean ± SEM) of the PFC Mu-burst were concomitantly detected in the dHPC (Figure 3C, top left). Hence, Mu-bursts could occur independently in these two structures. In the stress condition, the total number of Mu-bursts increased (Figure 3C, top right) in the dHPC (*p = 0.0156, Wilcoxon’s signed rank test) but not in the PFC (*p* = 0.8389, Wilcoxon’s signed rank test). Moreover, co-occuring Mu-bursts were detected in the PFC first and then in the dHPC, both at rest and under stress (**p = 0.0016 and ***p = 8e-15, respectively), with a median delay that was shorter at rest (0.20 s, Figure 3D, left) than under stress (0.32 s, Figure 3D, right). Hence, stress increased the occurrence of dHPC Mu-bursts, especially after the initiation of a PFC Mu-burst. We also detected Mu-burst in the PAR. These events could also be observed independently from the ones in the two neighboring structures (PAR and dHPC, Figure 4).

At a finer timescale (Figure 3D, top), phase shifts (derived from wavelet coherence) among simultaneous bursts appeared nearly constant during Mu-bursts (Figure 3D, bottom), consistent with a value of coherence around one, indicating a strong stability of the phase shift and a high covariation in amplitude. The dHPC PSD amplitude in the Mu-range was low in the absence of Mu-bursts, regardless of the stress or rest condition (Figure 3E, top). As expected, amplitude in the 7–12 Hz range increased strikingly during co-occurring Mu-bursts, an effect that was less pronounced when we focused on Mu-bursts occurring in single structures, e.g., only in the PFC or dHPC (Kruskal-Wallis test χ^2^ = 31.64; ***P_rest_ = 6.238e-7 and χ^2^ = 31.61; ***P_stress_ = 6.309e-7, Table 2 and Figure 3E, top). This profile was similar at rest or in stress condition. More intriguingly, phase-locking was also higher during Mu-bursts in one of the two structure (whatever the context) than in non-bursting episodes, suggesting synchronization processes between the PFC and dHPC even during “subthreshold” oscillations in the Mu range (Kruskal-Wallis test χ^2^ = 23.2 ***P_rest_ = 3.661 e-5 and χ^2^ = 23.65; ***P_stress_ = 2.961 e-5, Table 2 and Figure 3E, down). These results indicate that Mu-bursts can be generated independently in the PFC and dHPC, while being highly synchronized, and that stress affected the occurrence of Mu-bursts rather than the fine temporal relations between them. Acute stress increased the occurrence of Mu-bursts in both the PFC and dHPC, but impacted the total PSD in the dHPC only. Hence stress can affect the generation of Mu-bursts independently from the basal amplitude and phase of background oscillations in each structure.

**Table 2:** Impact of Mu-bursts on amplitude covariation and phase-locking value.

Group	Figure	Test	*P* value	*Post hoc*/correction	*P* value
**Adults rest:** Amplitude covariation	3E UP	Kruskal-Wallis ANOVA Table	χ^2^ = 31.64; ***P = 6.238e-7	Tukey’s honest significant Difference criterion	R1-R2: ***P< 0.0005 R1-R3: *P* = 0.0676 R1-R4: ***P< 0.0005 R2-R3: *P* = 0.08 R2-R4: *P* = 0.9715 R3-R4: *P* = 0.1553
**Adults stress:** Amplitude covariation	3E UP	Kruskal-Wallis ANOVA Table	χ^2^ = 31.61; ***P = 6.309e-7	Tukey’s honest significant Difference criterion	S1-S2: ***P< 0.0005 S1-S3: *P* = 0.2571 S1-S4: ***P = 0.0003 S2-S3: **P = 0.0061 S2-S4: *P* = 0.7691 S3-S4: *P* = 0.0995
**Adults rest:** Phase-locking value	3E Down	Kruskal-Wallis ANOVA Table	χ^2^ = 23.2; ***P = 3.661e-5	Tukey’s honest significant Difference criterion	R1-R2: ***P = 0.0003 R1-R3: **P = 0.0013 R1-R4: *P* = 0.0503 R2-R3: *P* = 0.7660 R2-R4: *P* = 0.4318 R3-R4: *P* = 0.8995
**Adults stress:** Phase-locking value	3E Down	Kruskal-Wallis ANOVA Table	χ^2^ = 23.65; ***P = 2.960e-5	Tukey’s honest significant Difference criterion	S1-S2: ***P< 0.0005 S1-S3: *P = 0.0119 S1-S4: *P = 0.0254 S2-S3: *P* = 0.2533 S2-S4: *P* = 0.3358 S3-S4: *P* = 1
**Aged rest:** Amplitude covariation	6E UP	Kruskal-Wallis ANOVA Table	χ^2^ = 15.16; **P = 0.0017	Tukey’s honest significant Difference criterion	R1-R2: **P = 0.0016 R1-R3: *P* = 0.9065 R1-R4: *P* = 0.1584 R2-R3: *P = 0.0209 R2-R4: *P* = 0.5218 R3-R4: *P* = 0.4971
**Aged stress:** Amplitude covariation	6E UP	Kruskal-Wallis ANOVA Table	χ^2^ = 5.41; *P* = 0.1444	N/A	N/A
**Aged rest:** Phase-locking value	6E Down	One-way ANOVA	*F*_(3,26)_ = 7.66; **P = 0.001	Tukey’s honest significant Difference criterion	R1-R2: **P = 0.0006 R1-R3: *P* = 0.059 R1-R4: *P* = 0.0960 R2-R3: *P* = 0.1663 R2-R4: *P* = 0.1576 R3-R4: *P* = 0.9995
**Aged stress:** Phase-locking value	6E Down	One-way ANOVA	*F*_(3,25)_ = 9.57; **P = 0.0003	Tukey’s honest significant Difference criterion	R1-R2: ***P = 0.0002 R1-R3: *P = 0.0265 R1-R4: *P* = 0.0759 R2-R3: *P* = 0.1745 R2-R4: *P* = 0.0770 R3-R4: *P* = 0.9678

Significant levels are presented with one to three asterisks (*P < 0.05, **P < 0.005, ***P < 0.0005).

### Interactions of Age and Stress on PFC and HPC Oscillations

**Figure 5:**
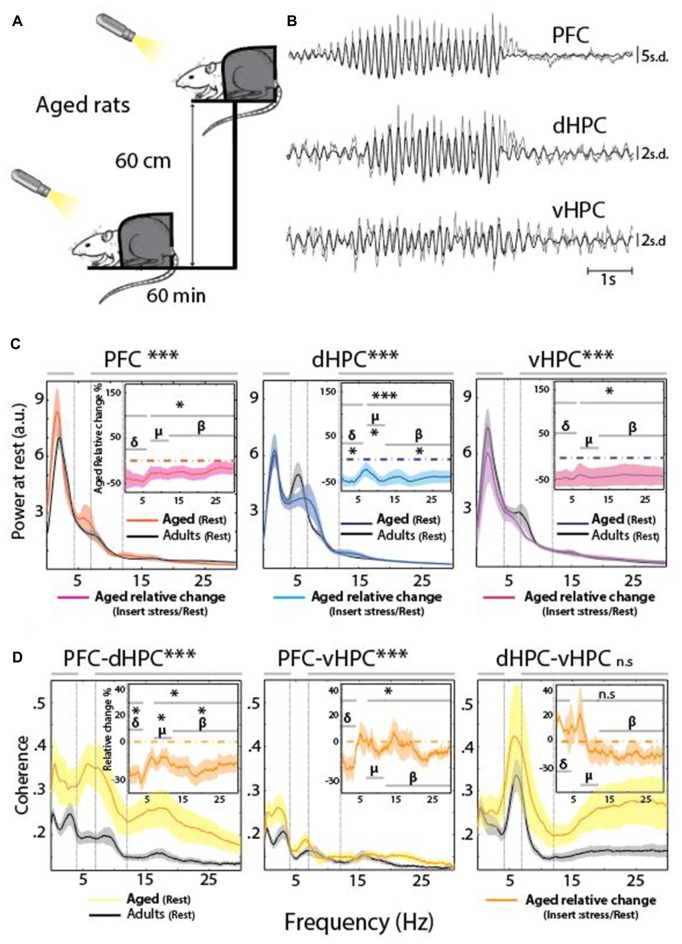
**(A)** Stress protocol (same as Figure 1) in aged rats. **(B)** Representative traces of the Z-scored EFP simultaneously recorded from the same animal in the PFC, dHPC and vHPC at rest. Raw traces are plotted in gray and filtered (Mu range) traces are overlaid in black. **(C)** Spectral analysis of the EFP recorded at rest for each structure and each group. Adults (black) Aged (color); red: PFC *n* = 10, blue: dHPC *n* = 9, purple: vHPC *n* = 5). Differences were observed between the average spectral properties of adults and aged rats at rest, whatever the structure (PFC: χ^2^ = 57.19; ***P < 0.001 Kruskal-Wallis test; dHPC: *F*_(2,63)_ = 132.45; ***P < 0.001 One-way ANOVA test; vHPC Kruskal-Wallis test χ^2^ = 41.61 ***P ≤ 0.001) and without specific frequency range. Inserts corresponded at the average relative changes under stress, expressed in percentage of variation. Stress in aged animal decreased the PSD amplitude in all brain structures (Friedman’s test: χ^2^ = 5.15, *P < 0.05 for the PFC; χ^2^ = 15.61, ***P ≤ 0.001 for the dHPC; χ^2^ = 5.53, *P < 0.05 for the vHPC) whatever the band after correction (see text) **(D)** Coherence for PFC-dHPC, PFC-vHPC and dHPC-vHPC at rest for each group (black: Adults; gold: Aged; PFC-dHPC *n* = 10, PFC-vHPC *n* = 5, dHPC-vHPC *n* = 5). Hippocampal-prefrontal synchrony at rest was significantly different between aged and adults rats, in the PFC-dHPC and PFC-vHPC (Kruskal-Wallis test: χ^2^ = 14.81 ***PCOH_PFC-dHPC_ < 0.001; χ^2^ = 24.26, ***PCOH_PFC-vHPC_ < 0.001). *Post hoc* (Holm’s Bonferroni) test showed that this difference did not implicate any specific band. Top-right insert: relative change after stress, expressed in percentage of variation. Horizontal dashed line at zero indicates no change. Shaded area indicated SEM. Stress decreased dramatically the coherence between PFC and dHPC in aged rats, for all frequency ranges taken separately (χ^2^ = 5.76, *P < 0.05 Friedman’s test followed by *post hoc* tests (Holm’s Bonferroni), *P_delta_ = 0.0166 paired-sample *t*-test; *P_mu_ = 0.0098 Wilcoxon’s signed rank test; *Pbeta = 0.0137 Wilcoxon’s signed rank test). Significant difference was also found in PFC-vHPC coherence without incrimination of a specific frequency band (χ^2^ = 4.98, *P < 0.05 Friedman’s test), but not in dHPC-vHPC (*F*_(1,24)_ = 0.02, *P* = 0.8766 Two-way ANOVA; ns *p* > 0.05; *p < 0.05; **p < 0.01, ***p < 0.001).

We then characterized the modifications of EFP spectral properties upon aging (late middle aged rats of 18 months, henceforth called “aged rats”, *n* = 10 for PFC and dHPC recordings, *n* = 5 for vHPC) and after stress exposure. Strikingly, at rest, aged rats exhibited Mu-bursts in the PFC and in the dHPC (Figures 5A,B), reminiscent of the Mu-bursts induced by stress in adults (Figures 1, 2), but no Mu-bursts in the vHPC (Figure 5B, see below for analysis), as in adults. Moreover, significant differences were observed: (i) between the average spectral properties of adults and aged rats whatever the structure but without specific frequency range (Figure 5C and Table 3); and (ii) in hippocampal-prefrontal synchrony in the PFC-dHPC and PFC-vHPC. *Post hoc* test showed that this difference did not implicate any specific band after statistical correction (Figure 5C, bottom).

**Table 3:** Baselines for age groups.

Group	Figure	Test	*P* value	*Post hoc*	Correction	*P* value
**PFC rest** Adults/Aged	5C Left	Kruskal-Wallis ANOVA Table	χ^2^ = 57.19; ***P = 3.814e-13	Delta: Two-sample *t*-test Mu: Wilcoxon rank sum test Beta: Wilcoxon rank sum test	Holm-Bonferroni: α/2< 0.025 α< 0.05 α/3< 0.0167	n/s *P*_Delta_: 0.3482 n/s *P*_Mu_: 0.4025 n/s *P*_Beta_: 0.1003
**dHPC rest** Adults/Aged	5C Middle	One-way ANOVA	*F*_(2,65)_ = 132.45; ***P = 2.711e-23	Delta: Two-sample *t*-test Mu: Two-sample *t*-test Beta: Two-sample *t*-test	Holm-Bonferroni: α< 0.05 α/3< 0.0167 α/2< 0.025	n/s *P*_Delta_: 0.9657 n/s *P*_Mu_: 0.1778 n/s *P*_Beta_: 0.4458
**vHPC rest** Adults/Aged	5C Right	Kruskal-Wallis ANOVA Table	χ^2^ = 41.61; ***P = 9.233e-10	Delta: Two-sample *t*-test Mu: Wilcoxon rank sum test Beta: Two-sample *t*-test	Holm-Bonferroni: α< 0.05 α/2< 0.025 α/3< 0.0167	n/s *P*_Delta_: 0.4550 n/s *P*_Mu_: 0.4421 n/s *P*_Beta_: 0.6993
**Rest**: Adults/Aged **Coherence_PFC-dHPC_**	5D Left	Kruskal-Wallis ANOVA Table	χ^2^ = 14.81; ***P = 0.0006	Delta: Two-sample *t*-test Mu: Two-sample *t*-test Beta: Wilcoxon rank sum test	Holm-Bonferroni: α< 0.05 α/3< 0.0167 α/2< 0.025	n/s *P*_Delta_: 0.0675 n/s *P*_Mu_: 0.0227 n/s *P*_Beta_: 0.0586
**Rest**: Adults/Aged **Coherence_PFC-vHPC_**	5D Middle	Kruskal-Wallis ANOVA Table	χ^2^ = 24.26; ***P = 5.387e-6	Delta: Two-sample *t*-test Mu: Wilcoxon rank sum test Beta: Two-sample *t*-test	Holm-Bonferroni: α/3< 0.0167 α< 0.05 α/2< 0.025	n/s *P*_Delta_: 0.0492 n/s *P*_Mu_: 0.8269 n/s *P*_Beta_: 0.1883
**Rest**: Adults/Aged **Coherence_dHPC-vHPC_**	5D Right	One-way ANOVA	*F*_(2,47)_ = 1.58; *P* = 0.2165	N/A	N/A	N/A

For multicomparisons tests (Two-way ANOVA or Friedman), significant levels (statistical significance was set at 5%) are presented with one to three asterisks (*P < 0.05, **P < 0.005, ***P < 0.0005) and for post hoc tests (Wilcoxon signed rank test or paired sample t-test) i.e. multicomparisons followed by Holm-Bonferroni corrections, by ordering the *p*-values *p*(1) < *p*(2) < *p*(3) and adjusting the significant level (1) α/3 < 0.0167,(2) α/2 < 0.025, (3) α < 0.05.

Stress in aged animal decreased the PSD amplitude in all brain structures (Table 1 and Figure 5C, insert), contrasting with adult rats where only the vHPC was affected (Figure 1C). This decrease was prominent in the dHPC whatever the band (*P_delta_ = 0.0078 Wilcoxon’s signed rank test; *P_mu_ = 0.0291 Paired sample *t*-test; *P_beta_ = 0.0118 Paired sample *t*-test). This might reflect a different reactivity of the vigilance state to stress in aged rats compared to adults. Furthermore, stress decreased dramatically the coherence between the PFC and dHPC in aged rats, for all frequency ranges taken separately (Figure 5D, insert right). This clearly contrasted with the stress-induced increase in coherence observed in adults (Figure 1D), and provides evidence for stress impacting cortical activity of aged and adult rats in an opposite fashion. Finally, significant difference was also found in PFC-vHPC coherence without incrimination of a specific frequency band, while coherence between the two parts of the hippocampus was not affected by stress.

**Figure 6:**
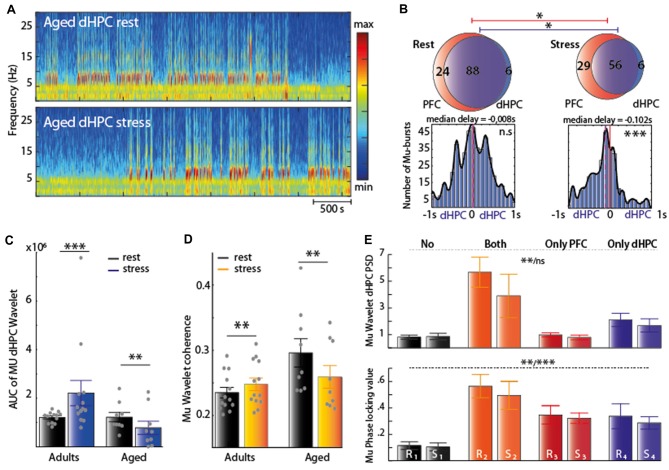
**(A)** Spectrogram of dHPC at rest (top) and under stress (bottom). Note the decrease of Mu-bursts under stress condition. **(B)** Top: Venn diagram illustrating the average occurrence of Mu-bursts in each structure and their co-occurrence and distribution of time lags between Mu-bursts onsets, from dHPC relative to PFC. Red line corresponds to the zero-lag and the purple line represents the median lag. Under stress condition, the total number of Mu-bursts decreased in the dHPC (*p < 0.05, Wilcoxon signed rank test) and in the PFC (*p < 0.05, paired sample *T*-test). At rest, there was on average no significant delay (median delay = −0.004 s) between PFC and dHPC bursts, while under stress, bursts were detected first in the dHPC (median delay = −0.0781 s, Wilcoxon signed rank test, ***p < 0.001). **(C,D)** AUC computed from the wavelet transform (left) and the wavelet coherence (right) from the 7–12 Hz range. In aged group, both decreased significantly under stress (black: rest; color: stress). Significant differences were found for AUC (Wilcoxson signed rank test *n* = 13 ***P_adults_ < 0.001 and paired sample *T*-test *n* = 9**P_aged_ < 0.001) and for coherence (paired sample *T*-test **P_adults_ < 0.01 and **P_aged_ < 0.01). **(E)** Top: square of the absolute value of the wavelet transform when no Mu-bursts occur (“No”), when Mu-bursts occur in both the PFC and dHPC (“both”) or only in one of the two structures (“only PFC” and “only dHPC) in two condition (R: rest; S: stress). dHPC PSD remained low in the absence of Mu-burst and during of occurring and co-occurring Mu-bursts only in the rest condition (Kruskal-Wallis test χ^2^ = 15.12, **P_rest_ < 0.05 and χ^2^ = 5.41, *P*_stress_ = 0.1444) Bottom: phase-locking value still remained significantly higher during co-occurring of the Mu-bursts at rest, and during PFC-occurring only (One-way ANOVA *F*_(3,23)_ = 7.66, **P_rest_ < 0.01 and *F*_(3,22)_ = 9.57 ***P_stress_ < 0.001). See table which indicated statistical difference between pairs.

We thus characterized the Mu-burst activity to assess the effect of stress in aged rats. Both at rest and under stress (Figure 6A), the vast majority of Mu-bursts co-occurred in the two structures PFC and dHPC (Figure 6B, top). Under stress, the total number of Mu-bursts decreased in the dHPC (*p = 0.0313 Wilcoxon signed rank test) and in the PFC (*p = 0.0298, paired sample *T*-test). However, at rest there was no significant delay between the PFC and dHPC bursts on average (Figure 6B, bottom left), while under stress, bursts were detected in the dHPC first (Figure 6B, bottom right). Hence temporal relations between Mu-bursts in the dHPC and PFC were inverted in adult and aged rats under stress. Finally, at rest, time-dependent spectrogram analysis suggested a high occurrence of Mu-bursts in aged rats in dHPC, similar to adult rats under stress (Figure 2A, right). The AUC from wavelet analysis confirmed a reduction under stress of dHPC Mu-bursts in aged rats (Figure 6C, **P_aged_ = 0.0057 Paired-sample *T-test)*. A similar decrease was observed in dHPC-PFC coherence (Figure 6D, **P_aged_ = 0.0075 paired sample *T*-test). Overall, both the occurrence of Mu-bursts in the PFC and dHPC, and the synchrony between these structures, were higher at rest in aged rats when compared to adults, and were differentially affected by stress (i.e., increased in adult vs. decreased in aged rats).

Paradoxically, compared to the adult group, dHPC PSD remained low only in the stress condition, during occurring (in a single structure) and co-occurring (PFC and dHPC) Mu-bursts (Kruskal-Wallis test χ^2^ = 15.16, **P_rest_ = 0.0017 and χ^2^ = 5.41, *P*_stress_ = 0.1444, Table 2 and Figure 6E, top). Interestingly at rest, phase-locking remained significantly higher exclusively when Mu-bursts appeared at the same time in both structures. Lastly, phase locking appeared to be significantly higher when these events were detected together, or only in the PFC, under stress (One-way ANOVA *F*_(3,23)_ = 7.66, **P_rest_ = 0.001 and *F*_(3,22)_ = 9.57, ***P_stress_ = 0.0003, Table 2 and Figure 6E, bottom).

These results appear fully consistent with the notions that aging is itself a stress factor (Morrison and Baxter, 2012; Lindenberger, 2014; Prenderville et al., 2015), and that aged individuals differently cope with stressful situations (Barrientos et al., 2012; Buechel et al., 2014).

### Effect of the Anxiolytic Diazepam on PFC and HPC Oscillations

**Figure 7:**
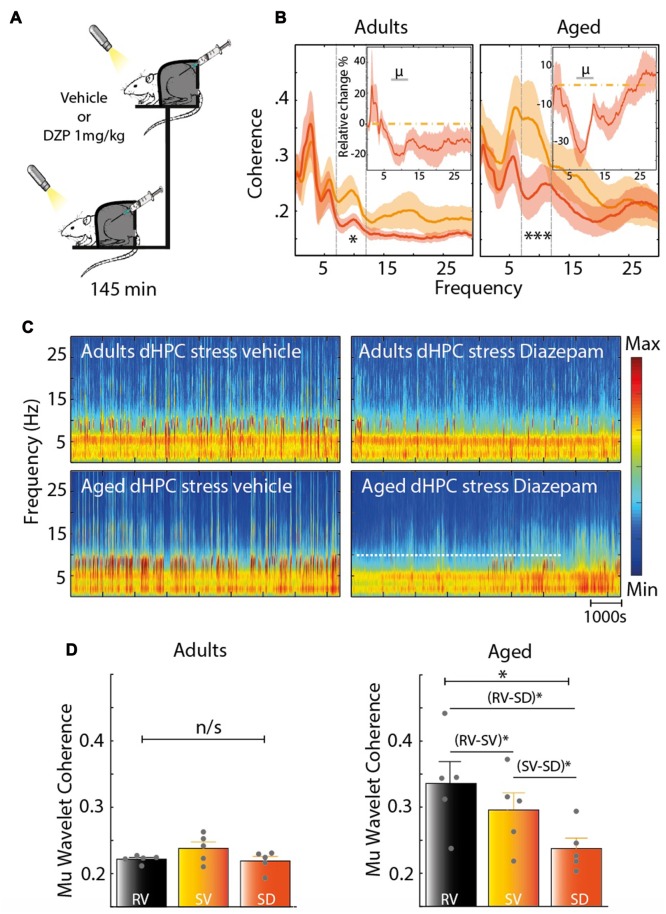
**(A)** Stress protocol (same as Figure 1) with an acute i.p injection of Diazepam (DZP; 1 mg/kg) vs. vehicle, in adult rats (*n* = 5) and aged rats (*n* = 5). **(B)** Coherence for PFC-dHPC in stress condition, under vehicle (orange) and under DZP (orange red). Top right insert: averaged relative change in coherence, expressed in percentage of variation. Horizontal dashed line at zero indicates no change. Shaded area indicates SEM. A significant decrease was found in the Mu band both for adult (left panel) and aged rats (right panel; *P_adults_ < 0.01; ***P_aged_ < 0.001, paired sample *T*-test). **(C)** Spectrogram of dHPC under stress for each group after a vehicle or DZP injection. Note the decrease of Mu-bursts under stress condition after an i.p injection of DZP. **(D)** PFC-dHPC coherence computed from the wavelet transform in the Mu range in three conditions (RV, rest vehicle; SV, stress vehicle; SD, Stress DZP (1 mg/kg)). Adults: COH_PFC-dHPC_: Kruskal-Wallis test χ^2^ = 1.63, *P* = 0.4431 for the PFC-dHPC coherence. Aged: Kruskal-Wallis test: χ^2^ = 6.02, *P < 0.05, followed by paired-sample *t*-test and Holm-Bonferroni correction *P_RV-SD_ = 0.0071, *P_RV-SV_ = 0.0102, *P_SV-SD_ = 0.0146 for the PFC-dHPC coherence.

**Figure 8:**
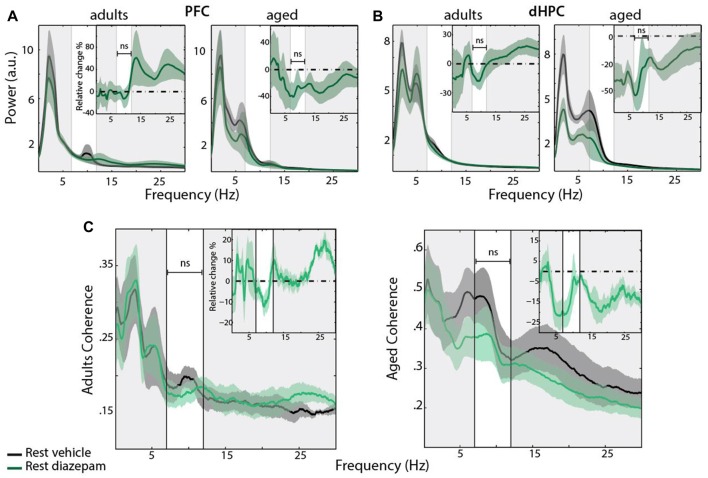
**(A,B)** Power spectrum density of PFC (left) and dHPC (right) EFP recorded at rest with an acute i.p injection of vehicle (black) vs. DZP 1 mg/kg (green), and the averaged relative change due to DZP (insert). Horizontal dashed line at zero indicates no change. Data are presented as mean ± SEM and shaded area indicates SEM. No significant change was observed in this condition, in both age groups. PFC: *P*_adults_ = 0.8491; *P*_aged_ = 0.2694, paired sample *T*-test; dHPC: *P*_adults_ = 0.4360; *P*_aged_ = 0.1473, paired sample *T*-test. **(C)** Coherence for PFC-dHPC (adults (left) and aged (right) rats) in rest condition, under vehicle (black) and DZP (green). Top right insert: averaged relative change in coherence, expressed in percentage of variation. Horizontal dashed line at zero indicates no change. Shaded area indicates SEM. No significant differences were found in Mu band in both group (*P*_adults_ = 0.2410; *P*_aged_ = 0.1159, paired sample *T*-test).

**Figure 9:**
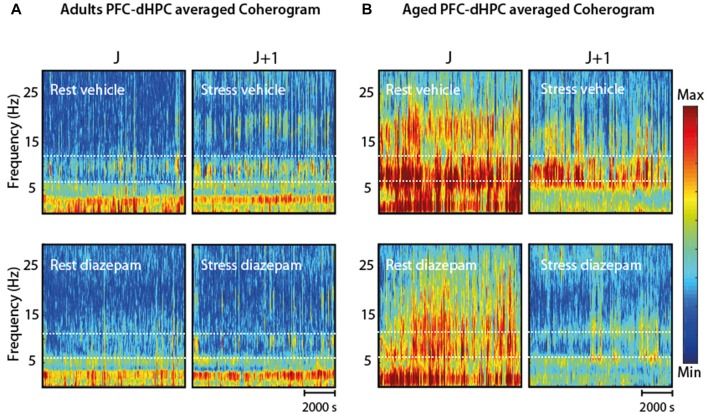
**(A)** Coherogram (PFC-dHPC coherence over time) in four conditions (RV; SV; rest-DZP; stress-DZP) for adult rats. Stress enhanced synchronization between PFC-dHPC in 7–12 Hz in the adult group compared to rest. Acute DZP injection reduced this synchrony at rest and under stress. **(B)** In aged rats, a global decrease (all frequency ranges) was observed under acute stress, but was less pronounced in the 7–12 Hz range. Conversely, DZP alleviated the coherence mainly in the 7–12 Hz. The combination of DZP and stress nearly abolished PFC-dHPC coherence for all frequency ranges.

Finally, we assessed how DZP, a widely-used anxiolytic, affects stress- and age-related changes on the dHPC-PFC coherence (145 min, *n* = 5 for each group; Figures 7A,B). In the stress condition, for each group, DZP decreased the coherence between the PFC and dHPC in the Mu-range, compared to saline (Figure 7B: left, Adults *P_mu_ = 0.0204, right, Aged ***P_mu_ = 0.0009). Time-dependent spectrograms of dHPC suggested that DZP abolished the increase in stress-related Mu-bursts (Figure 7C). In the adults group, DZP partially abolished the effects of stress on the coherence of hippocampal and prefrontal field potentials (Figure 7D, left). Contrarily, in aged rats, Mu-rhythms were reduced in the stress-DZP condition compared to the stress-vehicle (SV) condition and rest-vehicle (RV) condition (Figure 7). Furthermore, the effect of DZP on the Mu-rhythms seemed to be specific to stress: there was no changes in control non-stressed rats treated with DZP (Figure 8). Overall, these results suggest an efficient effect of DZP on adults (DZP abolishes stress effects), and an additive effect of DZP and stress in aged rats. These results are summarized in the average coherograms from the pharmacological protocol (Figure 9).

## Discussion

### Electrophysiological Markers of Stress in Immobile Rats

There is an ongoing debate on the implication of the different parts of the hippocampus in response to stress (Fanselow and Dong, 2010; Bannerman et al., 2014). While the vHPC is known to be directly implicated in anxiety-related processes through direct connections with the amygdala and bed nucleus of stria terminalis (Adhikari, 2014; Adhikari et al., 2015; Padilla-Coreano et al., 2016), the dHPC is believed to exert a role in contextual fear learning only (Bannerman et al., 2004; Fanselow and Dong, 2010). In most studies in rodents, analysis of hippocampal EFP focused on theta (4–12 Hz) oscillations, which in the dorsal part reveal prominent movement-dependent theta-rhythms (Buzsáki, 2002). Theta rhythms in the dHPC are generally of two types: type I theta, which is related to movement and is generated by the entorhinal cortex; and type II theta, which relates to alert immobility, arousal and anxiety and is generated by the medial septum and diagonal band of Broca (Vanderwolf, 1969; Kramis et al., 1975; Wells et al., 2013). Here we used a setup where rats could not move, enabling us to record type II theta and Mu rhythm, while avoiding contamination by type I theta, and we showed that dHPC rhythms were in fact modified by acute stress.

This is to our best knowledge the first report providing evidence that the dHPC PSD in adult rats significantly increased in the 7–12 Hz band under stress. Interestingly, these changes were exclusively caused by Mu-bursts rather than due to type II theta oscillation. These results were not observed in other studies, most probably because animals were free to move, e.g., in an elevated plus maze or an open field (Adhikari et al., 2010; Jacinto et al., 2013). In our experimental paradigm, changes in dHPC rhythms may be explained by animals being immobile (no theta I) or displaying a form of resignation to the long restraining time, with no escape possible (Balleine and Curthoys, 1991). Whatever the reason, these PSD increases in the 7–12 Hz range can be explained by fear experienced by rats when subjected to the EP, or by memorization of the stress context (but see “Interpretation of Mu Burst Events” Section for alternative interpretations).

Studies suggest that anxiety-like behaviors decrease, together with the activity of the vHPC circuit, when the environment becomes familiar. Likewise, we observed that vHPC PSD was globally desynchronized, probably due to a long exposure of the same environment, which is consistent with previous studies (Jacinto et al., 2013). Indeed, the environment in which the experiments took place was familiar (following habituation) to all groups of rats.

We found that, at rest, coherence in the 7–12 Hz range was very high between the two parts of the hippocampus (vHPC and dHPC). Coherence was also high between the PFC and the hippocampus but, unexpectedly, significantly higher with the dorsal than with the ventral part. Synchronizations were significantly increased by stress, yet only between the PFC and dHPC. These results are somewhat surprising considering the monosynaptic connections between the PFC and vHPC and the role of these structures in in anxiety (Verwer et al., 1997; Parent et al., 2010). Nevertheless, a strong coherence between the PFC and the dHPC is consistent with their anatomical relationship, which includes not only polysynaptic connections but also monosynaptic drive from the dorsal anterior cingulate cortex to the CA1/CA3 subfield (Rajasethupathy et al., 2015). Coherence analysis reflect functional cell assemblies, e.g., related groups of cells in distant brain structures with synchronized discharge to encode and store information (Battaglia et al., 2011). Hence, our results can be explained by a propagation of activity in the 7–12 Hz range from dHPC neurons, a crucial structure for the fast encoding of initial fear information, to the PFC, a structure with larger storage capacity, but slower learning, resulting into the consolidation of the fear memory.

### Modulation of Mu-Bursts by Age, Stress and Benzodiazepine

We detected in the PFC and dHPC (but not the vHPC) transient bursts of activity consisting in large amplitude oscillations in the 7–12 Hz frequency range, which we called Mu-bursts, and that seem associated with WT. These events were modulated in the dHPC by multiple factors, including age, stress and benzodiazepines. At rest, we observed a striking effect of the animal’s age, with more Mu-bursts in aged rats compared to adults, which is in agreement with previous studies (Aporti et al., 1986; Buzsáki et al., 1988; Ambrosini et al., 1997). This increased occurrence of Mu-bursts with age suggests these one may arise from the pathway specifically alters with aging. An important finding is that the 7–12 Hz rhythm of the dHPC was the frequency range the most impacted by stress, which suggests these events may be used as a biomarker for stress. Yet, stress acted on Mu-bursts in opposite fashion, i.e., increase vs. decrease, in adult and aged animals, respectively. Aged rats mays exhibit a hypersecreting HPA axis with increased corticotroprin release, and such glucocorticoid signaling might result in an exaggerated stress response (Buechel et al., 2014; Barrientos et al., 2015). This paradoxical result may alternatively be explained by the fact that, at rest, animals already exhibited different levels of Mu-burst activity.

In addition, we show that DZP decreased the occurrence of Mu-bursts, together with their co-occurrence, across all age in the stress condition. This is consistent with DZP acting as a positive allosteric modulator of GABA_A_ receptors, hence globally potentiating inhibition and inducing anxiolytic effects. However, because stress differently affect adult vs. aged animal, DZP overall reverted Mu-bursts occurrence and coherence in adult animals but almost abolished them in aged rats. Aging is associated with an altered composition in α1 and especially α5 subunits of GABA_A_ receptors (Yu et al., 2006; Schmidt et al., 2010). The GABAergic inhibition is less active in enhancing benzodiazepine binding in older animals, potentially due to the loss of functional GABA_A_ subunits (Calderini et al., 1981; Hoekzema et al., 2012). However, an increase in benzodiazepine binding sites was observed in aged rats, mainly in the hippocampus, striatum and cerebellum (Calderini et al., 1981). Hence, starting from a reduced inhibitory drive, acute administration of DZP may be more efficient in enhancing GABA_A_ function in old rats (Reeves and Schweizer, 1983). Our results suggest a definite effect of age on stress response and DZP administration. How this relates to alterations in oscillatory activity will be the focus of further work.

### Interpretation of the Mu-Burst Events

Mu-bursts, like spindles, have been traditionally observed in the cortex. Mu-bursts are associated with bursting in thalamic neurons and are believed to support distal communication between cortical areas and with the hippocampus (Fanselow et al., 2001). Although EFP reflect the activity of large groups of synapses, allowing identification of synchronous oscillatory activity within and across the brain areas, the exact anatomical origins of EFP must be tempered as voltage fluctuations can originate from volume conduction of distal signals. Our findings suggest that this was not the case here. First, we used a local reference (bipolar electrode) that minimizes electrical transfer (because common distal signals are subtracted), and provides the “intrinsic” EFP of the structure. Second, these bursts were found in adult rats in different combinations: only in the PFC, only in the dHPC, or in both structures. It is thus unlikely that dHPC bursts originate from the neocortex. Third, we also recorded from the parietal cortex, in the adult group and found that Mu-bursts could also be observed independently in two close neighboring structures (parietal and dHPC Figure 4). Therefore, our work, together with a previous study in the cerebellum (Hartmann and Bower, 1998), suggest that a much larger network of somatosensory structures (i.e., rather than the sole somatosensory cortex) may be flexibly involved in Mu-burst generation. The functional role of Mu-bursts is still a subject of debate in the literature: it has been hypothesized to reflect either a pathological (i.e., absence epilepsy, Inoue et al., 1990; Shaw, 2004, 2007) or physiological state (e.g., alertness or idling; Fanselow and Nicolelis, 1999; Fontanini and Katz, 2005). Even though we cannot definitely discard the hypothesis of an epileptic phenomenon, we believe that the effects of stress, age and anxiolytic we have observed on the 7–12 Hz bursts are of physiological background. First, similar “Mu rhythms” occur in 10%–30% of normal human subjects at rest (Nicolelis et al., 1995; Fontanini and Katz, 2005; Sakata et al., 2005; Tort et al., 2010) and have also been observed in cats (Guido and Weyand, 1995; Reinagel et al., 1999), guinea pigs (Edeline et al., 2000), rabbits (Swadlow and Gusev, 2002) and monkeys during periods of sensory processing (Ramcharan et al., 2000). These data suggest a functionally important and conserved physiological phenomenon. Second, we and others have observed that rats respond rapidly to stimuli during periods where Mu-bursts are detected (Vergnes et al., 1982; Fanselow et al., 2001) and these prominent oscillatory activities are invariably suppressed by movement, but not affected by eye opening (Buzsaki et al., 1988). This suggests that Mu-bursts do not reflect an epileptic state, which would be associated with an impaired sensory detection, but rather a “hyper alert” state of vigilance (Fanselow et al., 2001; Sobolewski et al., 2011) or, alternatively, an idling state during quiet immobility (Fontanini and Katz, 2005). Finally, Mu bursts have been associated with sensory-motor processing, which may provide an alternative interpretation of the Mu-burst modifications with age we observed. Animals were indeed isolated from any sensory inputs that might affect Mu oscillations, by placing them in an acoustically insolated chamber. Yet we cannot totally exclude the possibility that Mu oscillations reflected the sensory-motor processing of WT, rather than the level of alertness associated with WT. Along the same line, alterations of Mu oscillations in aged rats may have been caused by sensory impairment associated with aging. We did not quantify sensory function *per se*, even if aged rats were able to quickly detect sound.

Given that a high occurrence of Mu rhythms has been observed in humans during mind wandering (Braboszcz and Delorme, 2011; Kerr et al., 2013), we propose Mu-bursts in rats could correspond to a similar state of internal attention (Corballis, 2013a,b). Mind-wandering consists in disengaging from goal-oriented interactions with the external environment, with attention being directed inwardly to self-generated, stimulus-independent and task-unrelated thoughts. It is plausible that stress and age favor this mind state together with a disengagement from the environment (Killingsworth and Gilbert, 2010; Forster et al., 2015). Nonetheless, our study puts forward that stress-induced theta in the HPC and PFC is composed of Mu-bursts related to arousal. This provides an interesting electrophysiological framework to study the neurobiology of anxiety and anxiolytics, especially in the elderly.

## Author Contributions

CS, ES, MS, PF and JM designed the study. ST and BD performed the manufacture of bipolar electrodes and surgery. ST, BD and SD realized the experiment. ST, SD, JN and PF analyzed the data. JM provided the animal facility, the experimental sites and the electrophysiological equipment. ST, JN, AM and PF wrote the manuscript with inputs from SD and JM.

## Conflict of Interest Statement

MS and ES were employees of  Servier during a part of this work. The other authors declare that the research was conducted in the absence of any commercial or financial relationships that could be construed as a potential conflict of interest.
